# Durability Improvement of Pumice Lightweight Aggregate Concrete by Incorporating Modified Rubber Powder with Sodium Silicate

**DOI:** 10.3390/ma17040786

**Published:** 2024-02-06

**Authors:** Hailong Wang, Libin Shu, Kuaile Ma, Xingxing He

**Affiliations:** 1 College of Water Conservancy and Civil Engineering, Inner Mongolia Agricultural University, Hohhot 010018, China; 2021202060004@emails.imau.edu.cn; 2 Autonomous Region Collaborative Innovation Center for Integrated Water Resources and Water Environment Management in Inner Mongolia Section of Yellow River Basin, Hohhot 010018, China; 3 Department of Building Engineering and Technology, Vocational and Technical College of Inner Mongolia Agricultural University, Hohhot 014109, China; makuaile@imau.edu.cn; 4 State Key Laboratory of Hydroscience and Engineering, Tsinghua University, Beijing 100084, China; hexingxing16@mails.ucas.ac.cn

**Keywords:** sodium silicate, waste tire rubber powder, pumice lightweight aggregate concrete, freeze-thaw cycles, pore structure, fluid saturation

## Abstract

To improve the durability of pumice lightweight aggregate concrete applied in cold and drought areas, sodium silicate-modified waste tire rubber powder is used to treat the pumice lightweight aggregate concrete. The pumice lightweight aggregate concrete studied is mainly used in river lining structures. It will be eroded by water flow and the impact of ice and other injuries, resulting in reduced durability, and the addition of modified rubber will reduce the damage. The durability, including mass loss rate and relative dynamic elastic modulus of pumice lightweight aggregate concrete with different sodium silicate dosages and rubber power particle sizes, is analyzed under freeze-thaw cycles, and the microstructure is further characterized by using microscopic test methods such as nuclear magnetic resonance tests, ultra-depth 3D microscope tests, and scanning electron microscopy tests. The results showed that the durability of pumice lightweight aggregate concrete is significantly improved by the addition of modified waste tire rubber powder, and the optimum durability is achieved when using 2 wt% sodium silicate modified rubber power with a particle size of 20, and then the mass loss rate decreased from 4.54% to 0.77% and the relative dynamic elastic modulus increased from 50.34% to 64.87% after 300 freeze-thaw cycles compared with other samples. The scanning electron microscopy test result showed that the surface of rubber power is cleaner after the modification of sodium silicate, so the bonding ability between rubber power and cement hydration products is improved, which further improved the durability of concrete under the freeze-thaw cycle. The results of the nuclear magnetic resonance test showed that the pore area increased with the number of freeze-thaw cycles, and the small pores gradually evolved into large pores. The effect of sodium silicate on the modification of rubber power with different particle sizes is different. After the treatment of 2 wt% sodium silicate, the relationship between the increased rate of pore area and the number of freezing-thawing cycles is 23.8/times for the pumice lightweight aggregate concrete containing rubber power with a particle size of 20 and 35.3/times for the pumice lightweight aggregate concrete containing a particle size of 80 rubber power, respectively.

## 1. Introduction

Pumice is a special stone with a bubble shape formed after a magma eruption in full contact with the atmosphere. Pumice has the characteristics of porosity, soft texture, and low specific gravity. Using pumice as aggregate can solve the problems of self-weight, poor insulation, and sound insulation in ordinary concrete. The Soviet Union applied pumice concrete to practical engineering and observed it for 25 years, and the results show that the internal steel bar connection of pumice concrete is in good condition. In recent years, pumice concrete research has deepened, and pumice concrete is applied to the floor, roof board, floor beam, and other main construction parts. Pumice concrete is widely used in current civil construction, making it more economical and reasonable for people, but the use of pumice concrete in a large area needs theoretical support and practical verification. Its engineering application value is that it can be added to concrete to play a skeleton role, which is widely used in the production of pumice lightweight aggregate concrete (PLAC) [1]. When the pre-wet pumice replaces the coarse aggregate by 30~40%, the PLAC processed has the advantages of low density, good compressive strength, split tensile strength, and flexural strength, which obviously improves the dynamic performance of the interfacial transition zone (ITZ) [2,3,4,5]. In this case, the failure strain of PLAC is larger than that of ordinary concrete, which indicates pumice aggregate has good plastic deformation ability [1]. In addition, existing studies have shown that PLAC also has the advantages of low thermal conductivity, porous structure, and large elastic modulus [6,7], which can effectively isolate temperature and sound in engineering applications such as the channel lining structure and building insulation layer, etc. Wang et al. found that PLAC, as a hydraulic structure, is susceptible to fluid erosion and wear, ice impact, and repeated freeze-thaw cycles during its service, which resulted in serious deterioration of its mechanical stability and durability [8]. Therefore, the durability of PLAC against freeze-thaw cycles still needs to be further improved.

At present, numerous researchers are trying to adopt different modification methods to improve the durability of PLAC. For example, Mohamed Amin studied that adding polypropylene fiber and glass fiber to PLAC can change the failure behavior of concrete from brittle failure to ductile failure. [9,10]. Tung-Tsan Chen found that when 10% cement is replaced by mineral powder, both the compressive strength and elastic modulus of the PLAC specimen increase with the curing time [11]. Adding rubber particles to concrete can significantly improve the impact resistance and crack resistance of the concrete. Because rubber has good elasticity, when the concrete is impacted or vibrated, rubber particles can absorb and disperse part of the impact energy, thereby reducing the stress on the concrete structure and improving its durability and carrying capacity. Secondly, the application of rubber particles helps to improve the thermal insulation performance of concrete. Due to the low thermal conductivity of rubber, mixing it into concrete can effectively slow heat conduction, reduce the temperature difference between the inside and outside of the building, and improve the energy efficiency of the building. This is especially important in some temperature-sensitive building designs. M. Amiri found that the addition of rubber power (RP) can reduce the chloride ion migration rate in concrete, solving the utilization rate of waste tires and reducing pollution [12]. Jing Lv found that with the increase in RP, the fatigue life and strain of concrete generally increased under maximum and minimum loads [13]. The formation of RP is through waste tire mechanical processing and grinding; its surface is uneven and irregular, and it attaches a large number of impurities such as zinc stearate, etc. [14,15]. After the original rubber powder is added to the concrete, the porosity of the concrete is greatly increased, and then the water from the outside gradually erodes into the inside through the cracks around the rubber powder, which leads to the deterioration of its durability. Therefore, it is necessary to modify RP to improve the surface morphology of the particles.

Sodium silicate (SS) is a silicate that is soluble in water. Water glass has the characteristics of high bonding force, high strength, heat resistance, good acid resistance, and so on. Water glass and a hardening agent can be combined with ground filler or coarse aggregate to prepare heat-resistant mortar and concrete. Water glass is more widely used as an alkaline activator, used to stimulate the activity of fly ash, slag, etc. [16,17,18]. The research work on modifying water glass by adding new additives has also been favored by many scholars. SS is widely used as an alkaline activator in building materials. Cai found that adding SS increased the strength and compactness of Portland cement-based materials while reducing the pore size [19]. SS itself has the advantages of high viscosity and strength and can bond fly ash, cement, and other material particles, which will improve the strength and durability of concrete [20]. Researchers explained that the main components of rubber contain an acid carboxyl group, and the hydroxide ion in NaOH can replace the hydrogen ion on the functional group of acid, improving the bonding ability of the rubber powder-cement interface [21,22,23]. In addition, after NaOH treatment, the hydrophilicity of the rubber powder is increased, and the porosity of the cement-powder interface transition zone is reduced. Therefore, this study attempts to adopt SS to treat the RP, further improve the durability of PLAC modified by RP under freeze-thaw cycles, and provide some theoretical references for the development of hydraulic building concrete in northern China.

The application of fly ash to cement concrete has become a common practice. In many countries and regions, concrete containing fly ash is widely used in construction projects to improve project quality, reduce costs, and reduce the negative impact on the environment [2,24]. This trend is helping to promote the development of sustainable construction and engineering, pushing the construction industry in a more environmentally friendly and resource-efficient direction [25]. Fly ash can replace some cement because it contains active ingredients such as silicate and aluminate, which can play the role of cement cementing material. The fine particles of fly ash can fill the void between the cement particles and improve the fluidity and plasticity of concrete [26].

This work used SS to modify RP and then added the modified rubber power (MRP) to the pumice light aggregate concrete (PLAC) to prepare the concrete of RP-PLAC, further exploring the durability of RP-PLAC with different SS amounts and RP particle size under freeze-thaw cycles [27,28,29]. Some engineering properties of RP-PLAC, such as mass loss, dynamic elastic modulus, and microstructure properties under freeze-thaw cycles, are evaluated [30]. Microtests including scanning electron microscopy (SEM), nuclear magnetic resonance (NMR), and a super-distance-of-field 3D microscope, are carried out [4,31]. The microscopic mechanism of MRP improving the resistance to freeze-thaw cycles for PLAC is deeply revealed. This work can provide a reference for the extensive application of solid waste such as pumice and waste tires in civil engineering.

## 2. Test Materials and Scheme

### 2.1. Materials

In this study, ordinary Portland cement with a grade of 42.5 is obtained from Hohhot, Inner Mongolia. Some basic physical-chemical properties of materials are listed in Table 1. Fly ash can be supplied by a local power plant in Inner Mongolia and is also used in this experiment to replace 20% of the cement by mass, and its main chemical component is shown in Table 2. The modulus of SS (from Zhejiang in China) in this study is 3.3, and its performance index is shown in Table 3. Sodium lignosulfonate is used as a water-reducing agent (from Hebei in China), and its dosage is 1 wt% of the cementing material. The waste tires used in this study are mechanically ground first, and then the particles are ground into RP with particle sizes of 20 and 80, respectively. The apparent density and bulk density of the natural river sand used as fine aggregate are 2650 kg/m^3^ and 1465 kg/m^3^, respectively, and its fineness modulus is 2.5. The pumice used as coarse aggregate is collected from Hohhot City, Inner Mongolia Province, China, and its apparent density is 1569 kg/m^3^, bulk density is 706 kg/m^3^, and water absorption is 12.2%. Before the test, the pumice is crushed to a particle size of 16 mm~20 mm by a jaw crusher.

### 2.2. Material Pretreatment

**Figure 1:**
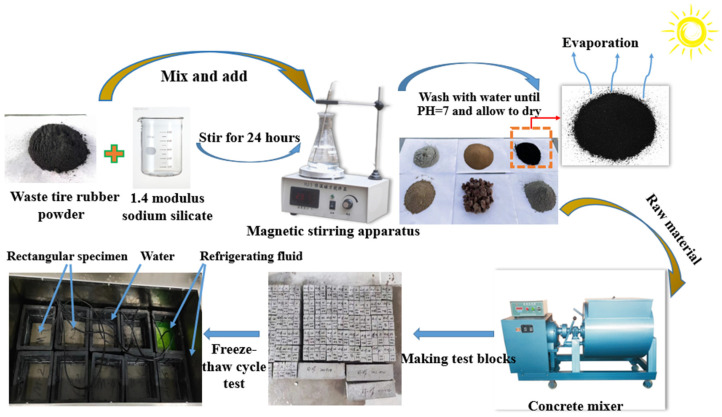
Process flow chart.

**Figure 2:**
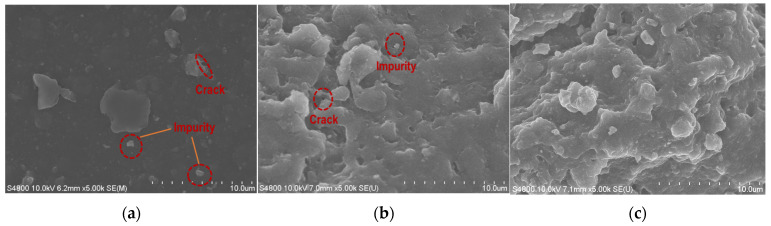
SEM image of rubber powder modification. (**a**) Virgin RP; (**b**) 2% SS MRP; (**c**) 4% SS MRP.

The modification process for RP is as follows: First, weigh a certain amount of NaOH (from Guangdong in China) powder and add it to the SS solution to adjust the modulus to 1.4. Secondly, soak an amount of RP with different particle sizes in SS solution with a mass ratio of 0 wt%, 1 wt%, 2 wt%, 4 wt%, 6 wt%, and 8 wt%, and then stir the solution in a magnetic stirrer for 24 h. Finally, clean the MRP with pure water until the PH of the solution is close to 7, and then dry the MRP in a natural environment for later use. The specific test steps are shown in Figure 1. The surface morphology changes of MRP particles are observed by the SEM (from Hitachi, Japan) test, and the results are shown in Figure 2.

As can be seen from Figure 2a, the surface of the virgin RP is relatively rough, and the diamond angle is obvious. Moreover, a large number of impurities can be observed attaching around the surface of RP particles [32]. According to Figure 2b, after 2 wt%SS is added, the RP surface becomes clean and the surrounding attachments are reduced, which is conducive to the interface combination of RP, cement material, and aggregate. This is because zinc stearate (C_17_O_35_COO)_2_Zn on the surface of RP reacts with NaOH in an acid-base reaction, and zinc stearate is the main cause affecting the reduction of binding force between RP and cement and aggregate. NaOH can remove the hydrophobic mixture on the RP surface and change the water contact angle of the RP [33]. The chemical equation is shown in Formulas (1) and (2).

(1) $$Na_{2}SiO_{3}+H_{2}O\to NaOH+H_{2}SiO_{3}$$

(2) $$(C_{17}O_{35}COO{)}_{2}Z_{n}+NaOH\to Na\left(C_{17}O_{35}COO\right)+Na_{2}(Z_{n}\left(OH{)}_{4}\right)$$

### 2.3. Test Scheme

The mix ratio is designed according to the ASTM C33/C33M-18 standard for concrete aggregates [34]. The mix design of PLAC is shown in Table 4. The size of the freeze-thaw cycle sample is 100 × 100 × 400 mm; each ratio requires the production of two parallel samples.

After curing for 28 days, the freeze-thaw cycle test is carried out. During the test, it adopts the rapid freeze-thaw method, with a cycle lasting about 2~4 h. During the freezing and melting tests, the center temperature of the specimen is controlled at −18 ± 2 °C and 5 ± 2 °C, respectively. After every 25 freeze-thaw cycles, the specimens are taken out for mass and dynamic elastic modulus tests.

The mass change of the specimen is one of the most significant indices to characterize the resistance to the freeze-thaw cycle. Before measuring the mass, the surface water of the specimen is wiped dry, the surface residue is removed, and it is then weighed on an electronic balance with a sensitivity of 0.01 kg. According to the test method in the standard of ASTM C215-14 [35], the dynamic elastic modulus of the specimen is tested, which is an important index to reflect the degree of internal compactness of the specimen, and the smaller the value of the dynamic elastic modulus, the greater the degree of damage and the lower the degree of internal compactness.

### 2.4. Microstructure Test Methods

In this study, MesoMR-60-type NMR (Newmax Electronic Technology Company from Nanjing, China)is used to analyze the pore structure of RP-PLAC. This instrument uses a permanent magnet as the core device. It needs to be preheated for 24 h during initial startup to achieve a temperature of 32 °C and a magnetic field stability of 300 Hz/h, ensuring the accuracy of the data. The magnetic field uniformity of the permanent magnet is 20 ppm, the probe coil diameter is 60 mm, the signal emission frequency is 300 w, the magnet strength is 0.55 T, and the resonance frequency generated by the H proton is 23.32 MHz. Through wet cutting, the diamond core drilling machine is used to take the core with a diameter of about 48 mm, and then the cylindrical test block with a height of about 50 mm is cut by the cutting machine. The prepared test block is saturated with water for 24 h in the vacuum saturation device under an air pressure of −0.1 Mpa. The relaxation time, peak area and proportion, porosity, bound fluid saturation, and free fluid saturation can be obtained [36]. A FEI Quanta FEG-type field emission scanning electron microscope (SEM) is used to observe the microstructural characteristics of the specimen after the freeze-thaw cycle. The acceleration voltage used in this study is 12 KV, the working distance is 6.4 mm, and the moving range of the sample platform is X = Y = 50 mm. The electron beam resolution is −1.4 nm/30 kV (SE), the electron beam voltage is 0.2–30 kV, and the amplification factor is 100–50,000×. The test sample is taken from the damaged part of the sample surface, and a complete plane can be used. Furthermore, a Lica-Z16APOA super-deep field 3D microscope is used to observe the surface morphology of the concrete specimen after the freeze-thaw cycle. The magnification range of the device is from 200× to 1500×, and the depth measurement accuracy is 2 μm. Observing the upper and lower outer surfaces of cylindrical specimens to form 3D images. Version 2.0 Leica Map software is used to construct 3D images of magnified 3D images by 30 times, forming cloud and contour maps. The sample to be measured is taken from the surface of the cuboid test block after a freeze-thaw cycle. 

## 3. Results and Analysis

### 3.1. Mass Loss Rate

During freeze-thaw cycles, the index of mass change rate (i.e., Rm) of the specimen can be calculated as Formula (3):

(3) $$Rm=\frac{m_{i}-m_{0}}{m_{0}}\times 100\%$$

where Rm represents the mass loss rate and mi represents the mass measured after the ith freeze-thaw cycle. m_0_ represents the mass measured before the freeze-thaw cycle. Rm = 0, <0, or >0 indicates that the mass of the corresponding specimen remains unchanged, decreases (i.e., loses), or increases, respectively, during the freeze-thaw cycles.

**Figure 3:**
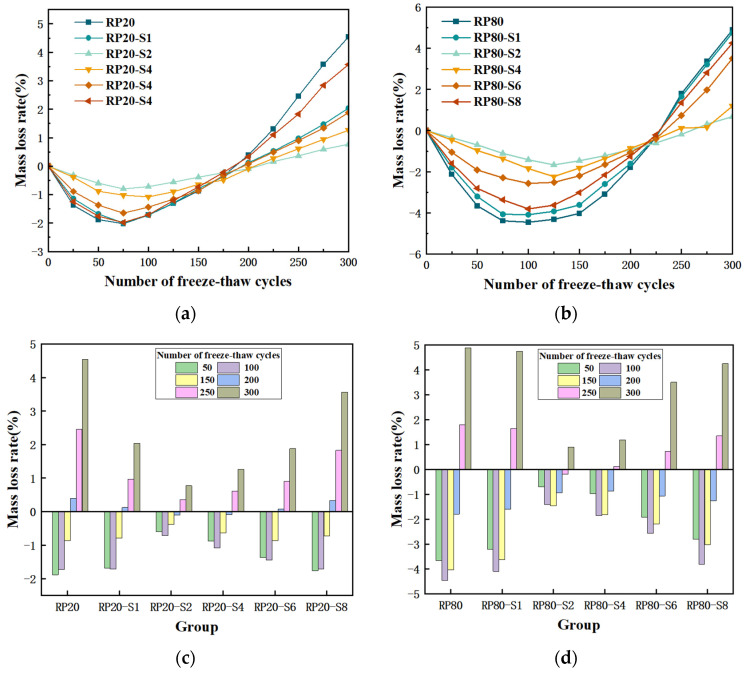
(**a**)The change trend of mass loss rate of the experimental group with 20 mesh RP as the change of the number of freeze-thaw cycles (**b**) The change trend of mass loss rate of the experimental group with 80 mesh RP as the change of the number of freeze-thaw cycles (**c**) Bar graph of the actual change of the mass loss rate with the number of freeze-thaw cycles in the 20 mesh RP experimental group (**d**) Bar graph of the actual change of the mass loss rate with the number of freeze-thaw cycles in the 80 mesh RP experimental group.

Figure 3 presents the Rm change of RP-PLAC specimens with different RP particle sizes and SS contents during the freeze-thaw cycles test. According to Figure 3a,c, it can be found that the Rm values of the specimens with a particle size of 20 RP are all less than 0 at the first 150 freeze-thaw cycles, which can infer that this stage is caused by the gradual infiltration of external free water into the internal pores of the specimen through the surface cracks. With the increase in the number of cycles, the water soaked the specimen repeatedly and gradually filled the pores of the whole sample, and finally, the water absorption rate basically reached saturation. Since then, the expansion pressure generated by the water inside the specimen during the freezing process has been greater than the tensile strength of the specimen itself, resulting in the frost-heave failure of the specimen, and the surface denudation has gradually aggravated. After 300 freeze-thaw cycles, the maximum and minimum values of Rm are 4.54% and 0.77%, respectively, and the corresponding specimens are RP20 and RP20-S2, indicating that modifying RP with a certain amount of SS could significantly improve the freeze-thaw cycle resistance of RP-PLAC. However, as the SS content increased to 4%, 6%, and 8%, the corresponding Rm values gradually increased to 1.26%, 1.88%, and 3.57%, respectively, indicating that excessive SS is unfavorable to the modification of RP. Therefore, when 2 wt%SS is used to modify RP with a particle size of 20, the corresponding RP-PLAC has the best durability during freeze-thaw cycles. 

Figure 3b,d present the Rm change of RP-PLAC specimens of RP with a particle size of 80 during freeze-thaw cycles. At the same Rm = 0, Group 80 is more resistant than Group 20. Group 20 Rm = 0 about 200 times, but Group 80 Rm = 0 about 250 times. According to the preliminary study of the team, it can be inferred that the rubber powder particles with smaller particle sizes have better-freezing resistance, but with the increase in the number of freeze-thaw cycles, the resistance in the later stage is poor. After 300 freeze-thaw cycles, the maximum and minimum values of Rm are 4.89% and 0.90%, respectively, and the corresponding specimens are RP80 and RP80-S2, thus their loss rate is the highest and the slowest. Later, as the SS content increased, the Rm values gradually increased to 1.19%, 3.51%, and 4.25%, respectively. Therefore, when 2 wt%SS is used to modify RP with a particle size of 80, the corresponding RP-PLAC has the best durability during freeze-thaw cycles. After 300 freeze-thaw cycles, the Rm of RP-PLAC of RP with a particle size of 80 is greater than that of RP-PLAC of RP with a particle size of 20; the maximum difference is about 2.3 times. This is because RP with a particle size of 80 has a large contact area with the concrete structure, resulting in the formation of internal pore channels. So the degree of water absorption is large, and the number of internal voids is large under freeze-thaw cycles, resulting in large expansion stress, so the damage degree of the specimen is large. The conclusion is the same as the above results. 

### 3.2. Relative Dynamic Elastic Modulus

**Figure 4:**
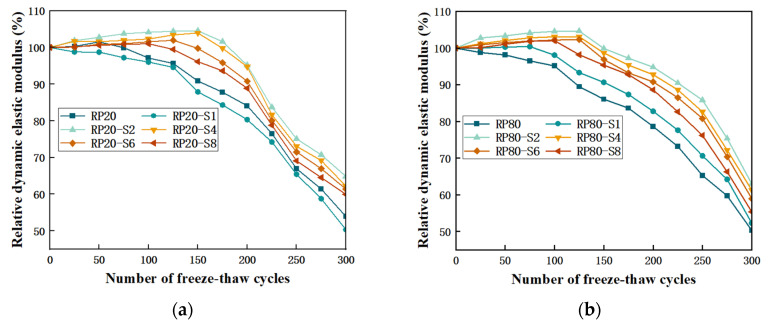

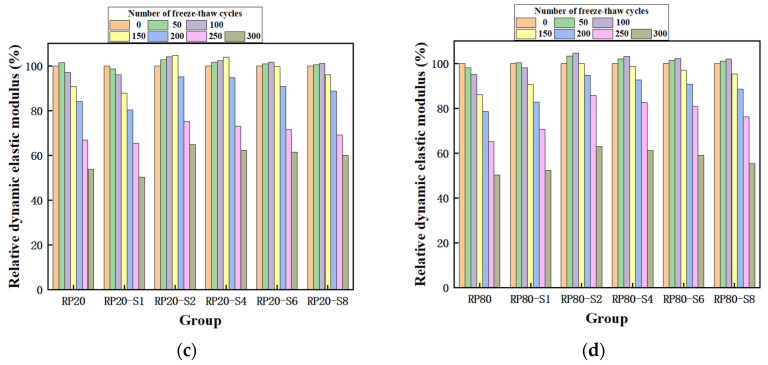
(**a**) The change trend of relative dynamic elastic modulus of the experimental group with 20 mesh RP as the change of the number of freeze-thaw cycles (**b**) The change trend of relative dynamic elastic modulus of the experimental group with 80 mesh RP as the change of the number of freeze-thaw cycles (**c**) Bar graph of the actual change of the relative dynamic elastic modulus with the number of freeze-thaw cycles in the 20 mesh RP experimental group (**d**) Bar graph of the actual change of the relative dynamic elastic modulus with the number of freeze-thaw cycles in the 80 mesh RP experimental group.

The dynamic elastic modulus of PLAC-RP specimens during freeze-thaw cycles is normalized as Formula (4):

(4) $$Re=\frac{e_{i}}{e_{0}}\times 100\%$$

where Re represents relative dynamic elastic modulus; $e_{i}$ represents the dynamic elastic modulus measured after the *i*th freeze-thaw cycle. $e_{0}$ represents the dynamic elastic modulus measured before freeze-thaw cycles. The calculation results of Re in the freeze-thaw cycles test are shown in Figure 4.

Figure 4a,c presents the change in Re values of RP-PLAC specimens of RP with a particle size of 20 during freeze-thaw cycles, generally (except RP20 and RP20-S1) showing a trend of first up and then down. Before 150 freeze-thaw cycles, the Re values present an upward trend. It can be inferred that in the early freeze-thaw cycles, the water infiltrated through cracks on the surface of the specimen will further react with cement and fly ash to generate new hydration products, and the Re is increasing. It can be found that the Re value of RP20-S2 is greater than that of other groups at different cycles, indicating that the PLAC with RP20-S2 has better durability, which is consistent with the result presented in Figure 3. After 150 freeze-thaw cycles, the hydration reaction basically ended, and then the Re values of the specimens gradually decreased as the number of freeze-thaw cycles increased. After repeated freezing and melting, the expansion stress of the pore water increased and finally accelerated the evolution of microcracks to macroscopic cracks, and the Re values present a downward trend. After 300 freezing-thawing cycles, the Re values of RP20 are 53.92%, and the maximum values of Re are 64.87% in RP20-S2, with the slowest decline rate. As the SS content increases, the Re values of RP20 gradually decrease, which are 62.22%, 61.40%, and 59.96%, respectively. Due to the increased alkali solution, part of C-S-H will dissolve, so the amount of SS is controlled at about 2 wt%.

Figure 4b,d present the Re values of RP-PLAC specimens of RP with a particle size of 80 during the freeze-thaw cycles test, and the Re values rise before 125 cycles. The contact area between RP with a particle size of 80 and cement slurry is large, thus forming a large number of pore channels, accelerating the entry of external water, and leading to a reduction in freeze-thaw cycles. After 300 cycles, the Re values of RP-PLAC of RP with a particle size of 80 are smaller than those of RP-PLAC of RP with a particle size of 20; the maximum Re values are about 1.08 times. In the two test groups, the Re values all have a declining stage, and there are two reasons for the decline: first, RP is dispersed in PLAC, forming pores around RP with high hydrophobicity; second, the Re values of RP are small themselves, forming a weak area around it. From the Re values after 300 cycles, the RP20-S2 still has the best resistance to freeze-thaw cycle damage, which is consistent with the above conclusions.

### 3.3. Pore Structure Characteristics 

**Figure 5:**
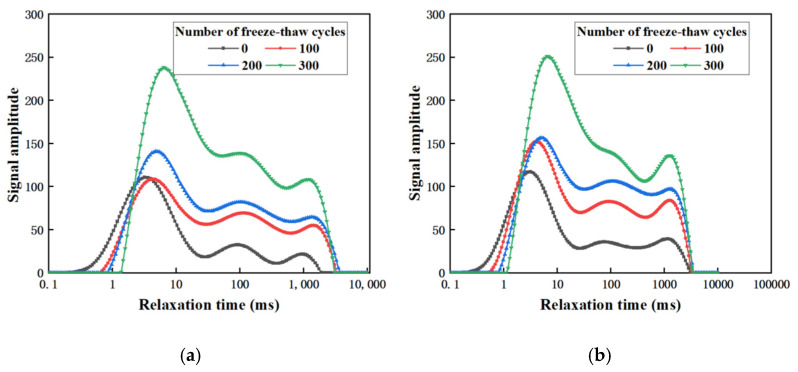
Pore structure evolution of specimen after freeze-thaw cycle. (**a**) *T*_2_ spectrum curve of RP20-S2; (**b**) *T*_2_ spectrum curve of RP80-S2.

NMR technology is widely used to detect pore water content and pore volume in concrete materials [37]. The NMR data of the optimal groups of RP20-S2 and RP80-S2 are measured and analyzed after 0, 100, 200, and 300 freeze-thaw cycles, respectively, as shown in Figure 5. The T_2_ spectrum is composed of different relaxation times and corresponding amplitudes, which is a comprehensive reflection of rock information such as pore structure and fluid composition. It is related to pore radius and pore number. The relaxation time represents the pore size; the larger the value, the larger the pore radius [38]. There are three kinds of relaxations in the concrete, and the sum of the reciprocal of the three relaxation states is equal to the reciprocal of the transverse relaxation time. The formula is shown in (5).

(5) $$\frac{1}{T_{2}}=\frac{1}{T_{2F}}+\frac{1}{T_{2S}}+\frac{1}{T_{2D}}$$

where *T*_2_ is the time required for energy exchange when the energy levels of the core alternate, that is, the transverse relaxation time of the porous fluid (ms); *T*_2*F*_ is the transverse relaxation time of infinite pore fluid (ms); *T*_2S_ is the surface relaxation time (ms); and *T*_2*D*_ is the transverse relaxation time of pore fluid caused by diffusion under a gradient magnetic field (ms).

(6) $$\frac{1}{T_{2}}=\rho_{2}\left(\frac{S}{V}\right)=\rho_{2}\left(\frac{3}{r}\right)$$

where $\rho_{2}$ is the surface relaxation strength (μm/s), we take the empirical value *ρ*_2_ = 5 (μm/s); *S* is the pore surface area (μm^2^); *V* is the pore volume (μm^3^); $\frac{S}{V}$ is the ratio of pore surface area to volume (μm^−1^).

According to Figure 5, the relaxation time of both groups moves to the right with the increase in the number of freeze-thaw cycles, indicating that the internal pores become larger, and the evolution trend from small pores to large pores is obvious. The *T*2 spectral area usually corresponds to the number of internal pores. The larger the area, the greater the number of pores in the specimen. It can be seen that the curves in Figure 5a,b all present a “three-peak” structure. With the increase in freeze-thaw cycles, not only does the pore radius increase, but also the number of pores. The area of the RP80-S2 increases with the cycles as follows: 4930.99, 6325.997, 8682.777, and 12073.68; the growth rate is 23.8/time; and the area of the RP20-S2 increases with the cycles as follows: 6182.978, 8050.055, 11870.5, and 16776.62; the growth rate is 35.3/times. 

**Figure 6:**
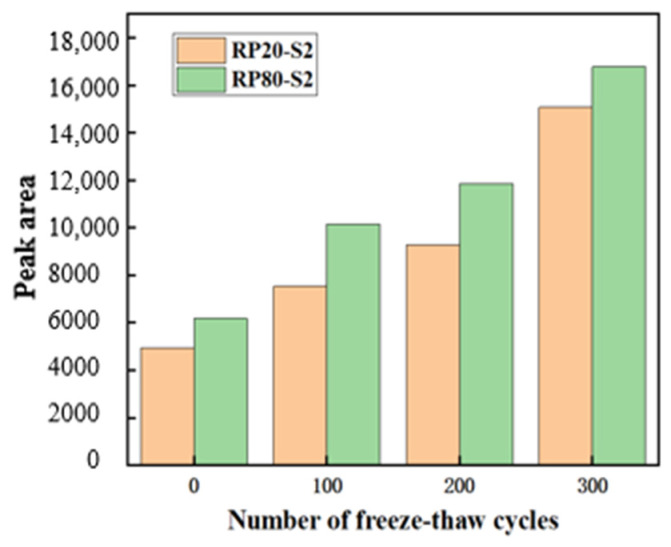
Pore area of specimens after freeze-thaw cycles.

**Figure 7:**
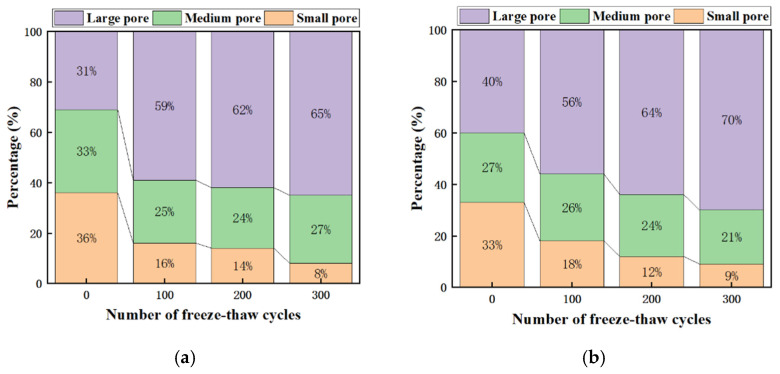
The pore area and proportion after freeze-thaw cycles. (**a**) RP20-S2; (**b**) RP80-S2.

According to the pore classification of concrete by Wu Zhongwei [39], the pores are classified as small pore (<0.05 μm), medium pore (0.05~0.20 μm), and large pore (>0.20 μm), and the pore percentage of RP20-S2 and RP80-S2 is derived. In terms of pore area and growth rate, as shown in Figure 6 and Figure 7, as the number of cycles increases, small pores gradually deteriorate into large pores, and the RP80-S2 has larger pores than the RP20-S2, indicating that the RP20-S2 has better resistance to freeze-thaw cycles than the RP80-S2.

**Figure 8:**
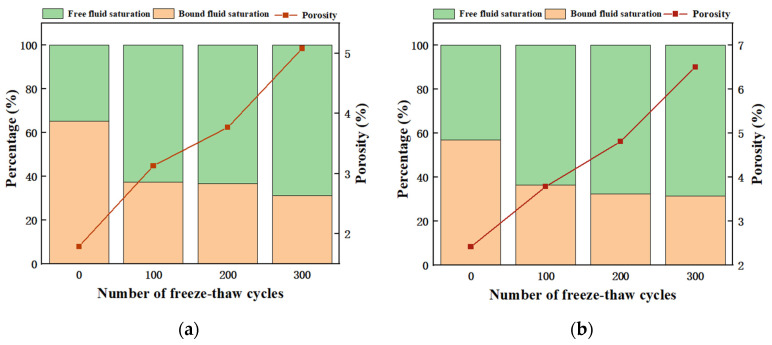
The pore water distribution after the freeze-thaw cycles. (**a**) RP20-S2; (**b**) RP80-S2.

Figure 8 presents the porosity, free water saturation, and bound water saturation of the optimal group (RP20-S2, RP80-S2), which can be directly obtained during the test process. Porosity has an important influence on the frost resistance of concrete [40]. Bound water saturation refers to the ratio of the pore volume of non-mobile-bound water to the total pore volume in the concrete. As the number of cycles increased, the small and medium-sized pores in the specimen gradually decreased, and the proportion of free water increased while the proportion of non-flowing-bound water decreased. Porosity is the main factor that characterizes the internal microstructure of concrete. After 300 freeze-thaw cycles, the maximum porosity of the RP20-S2 and RP80-S2 is 5.083% and 6.504%, respectively. The increase in porosity in RP20-S2 is lower than that in RP80-S2 during 100–200 cycles. This is due to the fact that the particle size of RP with a particle size of 20 is larger than that of RP with a particle size of 80, and there is less chance to blend with the cement paste, so there are more opportunities for hydration reactions with the cement paste after the water enters, making the structure in a dense state. On the contrary, RP80-S2 is fully mixed with cement paste, with many pore channels. RP blocks the hydration of external water and cement, resulting in a stable increase in porosity.

### 3.4. Microstructure Analysis

#### 3.4.1. Super Depth of Field 3D Microscope Analysis

**Figure 9:**
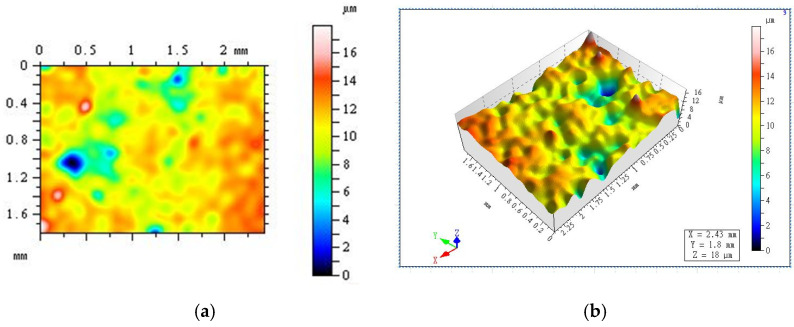

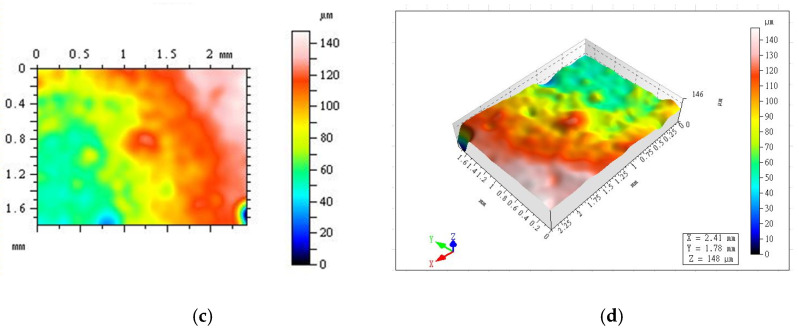
Super depth of field 3D of RP20-S2 before and after freeze-thaw cycles. (**a**) Apparent height difference map; (**b**) 3D map; (**c**) Apparent height difference map; (**d**) 3D map.

Figure 9 shows the super-depth field 3D images of the specimen surface in RP20-S2 before and after 300 freeze-thaw cycles. The surface height difference between specimens is represented according to the different colors in the images. The blue areas present the holes and cracks in the concrete surface, while the red areas correspond to the raised parts. It can be seen from (a) that the red area takes up a large part of the figure while the blue area takes up a small part, indicating that there are a lot of small convex parts on the surface of the specimen but few holes and cracks, and the maximum surface elevation difference is 17.7 μm. After 300 freeze-thaw cycles, as shown in (c), the blue area is larger, indicating that the holes and cracks on the concrete surface gradually become larger and deeper with the increase in the number of freeze-thaw cycles, and the maximum surface height difference is 110.21 μm. 

#### 3.4.2. SEM Analysis

**Figure 10:**
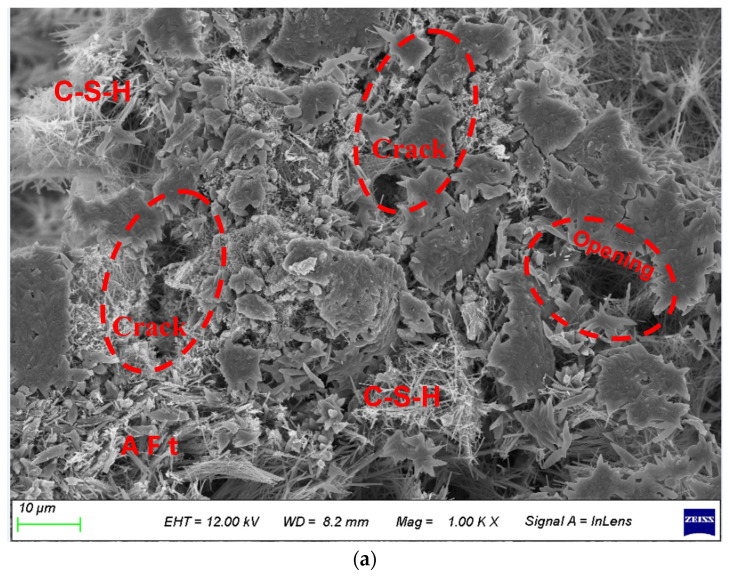

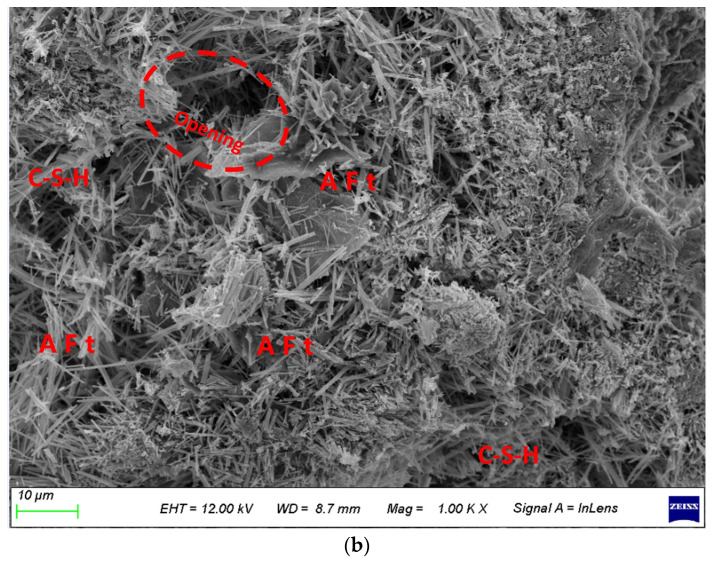
SEM images of 200 freeze-thaw cycles of RP20 and RP20-S2. (**a**) RP20 freeze-thaw cycle 300 times; (**b**) RP20-S2 freeze-thaw cycle 300 times.

The SEM test results of RP20 and RP20-S2 are shown in Figure 10. It is obvious that there are many cracks on the surface of RP20 (Figure 10a), and only a small amount of typical hydration products is found. After 300 cycles, many large pores and deep cracks appear, and some small cracks are gradually destroyed into large cracks. This is due to some cracks and poor bonding between the RP particle and the cementing material and aggregate. According to Figure 10b, there is a large amount of acicular and reticular calcium silicate hydrate (C-S-H) gel in RP20-S2, while a lot of rod-shaped ettringite (AFt) is interspersed between C-S-H and interwoven with each other to enhance the structural integrity. It proves that the MRP with 2 wt%SS improves the interface bonding performance of concrete. In conclusion, the internal compacting degree of RP20-S2 is better than that of RP20, which explains the large dynamic elastic modulus of RP20-S2. Therefore, the addition of 2 wt%SS can significantly improve the freeze-thaw cycle resistance of the specimen.

## 4. Conclusions

This study focuses on using sodium silicate (SS) to modify rubber powder (MRP) of different particle sizes (RP with particle sizes of 20 and 80), then the MRP is added to pumice light aggregate concrete (PLAC), and the durability of RP-PLAC is verified by the freeze-thaw cycles test. The mass loss rate (Rm) and the relative dynamic elastic modulus (Rm) of RP-PLAC are measured and analyzed. Some microtests, including a super-deep-field 3D microscope and SEM, are performed to reveal the mechanism of durability enhancement of RP-PLAC under freeze-thaw cycles. The following conclusions are drawn:

(1) According to the SEM image of modified rubber, the surface of rubber modified with 2 wt% sodium silicate is cleaner, which is due to the chemical reaction of sodium hydroxide and zinc stearate to remove some impurities. As the amount of SS increases, the modified rubber surface becomes cleaner, which is conducive to the combination of rubber particles and cement paste.
(2) The mass loss rate generally showed a trend of decreasing first and then increasing. The decreasing stage was due to the repeated entry of water into the sample, and the water absorption rate of the sample reached saturation; the rising stage was due to the freezing and failure of the sample and the spalling of surface materials. The durability of RP-PLAC with a particle size of 80 is better than that of RP-PLAC with a particle size of 20.
(3) After 300 freeze-thaw cycles, 2 wt% sodium silicate can significantly improve the durability of RP-PLAC. The addition of RP with a particle size of 80 to the RP-PLAC prevents external water from entering the sample and delays the transformation of the surface structure from small cracks to large cracks. According to SEM images, C-S-H, with its large number of fiber structures and network structures, prevents the deterioration of structures.
(4) The results of the 3D microscope imaging test show that modified rubber used in PLAC can significantly reduce the relative height difference of the specimen surface during freeze-thaw cycles by about 83.9%, thus confirming that MRP used in PLAC can improve the resistance of concrete to freeze-thaw cycles.

## Figures and Tables

**Table 1:** Physical and chemical properties of cement.

Specific Surface Area (m^2^/kg)	Initial Setting Time (min)	Final Setting Time (min)	Stability	SiO_2_ Mass Fraction (%)	Loss on Ignition (%)	Compressive Strength (MPa)		Rupture Strength (MPa)	
						3 d	28 d	3 d	28 d
350	150	240	Qualified	22.12	1.02	25.6	54.8	5.3	8.3

**Table 2:** Chemical component of fly ash and cement.

Element	SiO_2_	Al_2_O_3_	Fe_2_O_3_	MgO	CaO	Other	Loss of Ignition
Fly ash	58.27	24.35	5.43	0.76	6.11	5.08	3.1
Cement	21.29	5.20	4.11	0.49	63.50	5.41	<5

**Table 3:** Sodium silicate performance index.

Module	Na_2_O (%)	SiO_2_ (%)	Density (20 °C) (g·cm^−3^)	Water Insoluble (%)
3.3	8.3	26.5	1.385	0.02

**Table 4:** Mix ratio design of PLAC.

Sample	Cement	Fly Ash	Sand	Pumice	Water	Sodium Silicate (%)	Rubber Size	Rubber Content (%)	Water Reducing Agent (%)
	(kg/m^3^)								
RP20	408	102	785	546	204	0	20	3	1
RP80	408	102	785	546	204	0	80	3	1
RP20-S1	408	102	785	546	204	1	20	3	1
RP20-S2	408	102	785	546	204	2	20	3	1
RP20-S4	408	102	785	546	204	4	20	3	1
RP20-S6	408	102	785	546	204	6	20	3	1
RP20-S8	408	102	785	546	204	8	20	3	1
RP80-S1	408	102	785	546	204	1	80	3	1
RP80-S2	408	102	785	546	204	2	80	3	1
RP80-S4	408	102	785	546	204	4	80	3	1
RP80-S6	408	102	785	546	204	6	80	3	1
RP80-S8	408	102	785	546	204	8	80	3	1

Notes: Conversion of particle size to millimeters: Diameter = 25.4/particle size × 0.65 (1 inch = 25.4 mm). So, a particle size of 20 has a diameter of 0.85 mm, and a particle size of 80 has a diameter of 0.2 mm.

## Data Availability

Data are contained within the article.
